# QuickStats

**Published:** 2013-06-21

**Authors:** Michael Albert, Jill J. Ashman

**Figure:**
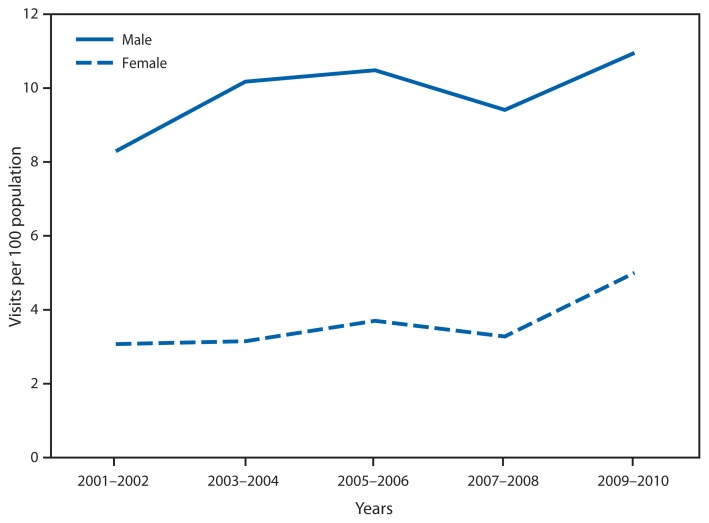
Rate of Ambulatory-Care Visits* for Attention Deficit Hyperactivity Disorder (ADHD)^†^ by Persons Aged ≤18 Years, by Sex — United States, 2001–2002 to 2009–2010 * Visits to hospital outpatient departments and physician offices per 100 population. Rates were calculated using U.S. Census Bureau 2000–based postcensal civilian population estimates. ^†^ Defined as having a first-listed diagnosis of 314.00 or 314.01, as coded according to the *International Classification of Diseases, Ninth Revision, Clinical Modification*.

From 2001–2002 to 2009–2010, the ambulatory-care visit rate for ADHD for females aged ≤18 years increased by 63%, from 3.1 to 5.0 visits per 100 population. Over the same period, the change in the visit rate for males did not follow a consistent pattern; in 2009–2010, the visit rate for males was 11.0 per 100. Throughout the period, males were more likely than females to have an ambulatory-care visit for ADHD.

**Sources:** National Ambulatory Medical Care Survey 2001–2010. Available at http://www.cdc.gov/nchs/ahcd.htm.

